# [2,6-Bis(6-methyl­quinolin-2-yl)pyridine-κ^3^ *N*,*N*′,*N*′′]dichloridoiron(II)

**DOI:** 10.1107/S1600536810037049

**Published:** 2010-09-25

**Authors:** Xiao-Ping Li, Jian-She Zhao, Seik Weng Ng

**Affiliations:** a Department of Chemistry, Shaanxi Key Laboratory for Physico-Inorganic Chemistry, Northwest University, Xi’an 710069, People’s Republic of China; b Department of Chemistry, University of Malaya, 50603 Kuala Lumpur, Malaysia

## Abstract

In the mol­ecule of the title compound, [FeCl_2_(C_25_H_19_N_3_)], the three N atoms span the axial–equatorial–axial sites of the trigonal-bipyramidal coordination polyhedron; the geometry of the Fe^II^ atom is 32% distorted from trigonal-bipyramidal (towards square-pyramidal along the Berry pseudorotation pathway). One of the Cl atoms is disordered over two positions in a 0.938 (11):0.062 (11) ratio. Inter­molecular C—H⋯Cl hydrogen bonding occurs in the crystal structure.

## Related literature

For the synthesis of the *N*-heterocyclic ligand, see: Buu-Hoi *et al.* (1965 ▶). For a related structure, see: Li *et al.* (2010 ▶).
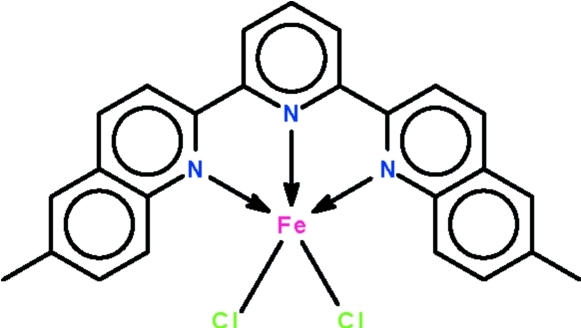


## Experimental

### 

#### Crystal data

[FeCl_2_(C_25_H_19_N_3_)]
*M* *_r_* = 488.18
Triclinic, 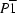

*a* = 9.6228 (7) Å
*b* = 10.2558 (8) Å
*c* = 10.7324 (8) Å
α = 94.352 (1)°
β = 95.481 (1)°
γ = 96.121 (1)°
*V* = 1044.35 (14) Å^3^
*Z* = 2
Mo *K*α radiation
μ = 1.00 mm^−1^
*T* = 100 K
0.30 × 0.10 × 0.05 mm

#### Data collection

Bruker SMART APEX diffractometer
Absorption correction: multi-scan (*SADABS*; Sheldrick, 1996 ▶) *T*_min_ = 0.754, *T*_max_ = 0.952
9910 measured reflections
4757 independent reflections
3954 reflections with *I* > 2σ(*I*)
*R*_int_ = 0.024

#### Refinement

*R*[*F* ^2^ > 2σ(*F* ^2^)] = 0.035
*wR*(*F* ^2^) = 0.108
*S* = 1.05
4757 reflections
292 parameters
7 restraints
H-atom parameters constrained
Δρ_max_ = 0.54 e Å^−3^
Δρ_min_ = −0.44 e Å^−3^

### 

Data collection: *APEX2* (Bruker, 2009 ▶); cell refinement: *SAINT* (Bruker, 2009 ▶); data reduction: *SAINT*; program(s) used to solve structure: *SHELXS97* (Sheldrick, 2008 ▶); program(s) used to refine structure: *SHELXL97* (Sheldrick, 2008 ▶); molecular graphics: *X-SEED* (Barbour, 2001 ▶); software used to prepare material for publication: *publCIF* (Westrip, 2010 ▶).

## Supplementary Material

Crystal structure: contains datablocks global, I. DOI: 10.1107/S1600536810037049/xu5032sup1.cif

Structure factors: contains datablocks I. DOI: 10.1107/S1600536810037049/xu5032Isup2.hkl

Additional supplementary materials:  crystallographic information; 3D view; checkCIF report

## Figures and Tables

**Table 1:** Selected bond lengths (Å)

Fe1—N1	2.2386 (19)
Fe1—N2	2.103 (2)
Fe1—N3	2.2523 (19)
Fe1—Cl1	2.3636 (8)
Fe1—Cl2	2.2748 (7)

**Table 2:** Hydrogen-bond geometry (Å, °)

*D*—H⋯*A*	*D*—H	H⋯*A*	*D*⋯*A*	*D*—H⋯*A*
C8—H8⋯Cl2^i^	0.95	2.70	3.561 (2)	151
C17—H17⋯Cl1^ii^	0.95	2.73	3.538 (4)	144

Symmetry codes: (i) 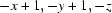; (ii) 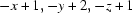.

**Fig. 1.**
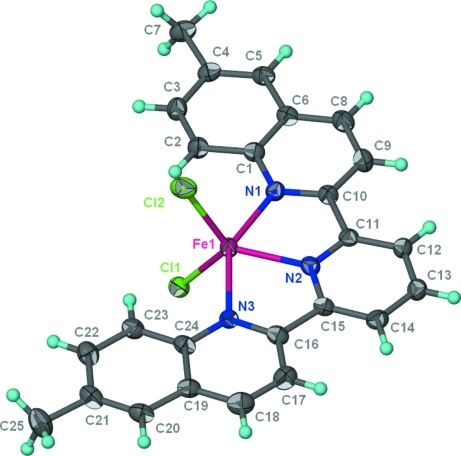
Thermal ellipsoid plot (Barbour, 2001) of FeCl2(C25H19N3) at the 70% probability level; hydrogen atoms are drawn as spheres of arbitrary radius. The disorder is not shown.

## supplementary crystallographic information

### Comment

A recent study reported the chromium(III) chloride adduct of 2,6-bis(*p*-bromphenylimino)pyridine; the *N*-heterocycle chelates to the metal atom in a terdentate manner (Li *et al.*, 2010). Bis[2'-(6-methylquinolinyl)]pyridine has a similar set of donor sites capable of binding in this manner, as demonstrated in the present iron dichloride adduct (Scheme I, Fig. 1 and Table 1). In the molecule of FeCl_2_(C_25_H_19_N_3_), the three N atoms span the axial–equatorial-axial sites of the trigonal bipyramidal coordination polyhedron; the geometry of Fe is 32% distorted from the trigonal bipyramid along the Berry pseudorotation pathway. Intermolecular C—H···Cl hydrogen bonding occurs in the crystal structure (Table 2).

### Experimental

The ligand was synthesized by using a literature procedure (Buu-Hoi *et al.*, 1965).

Bis[2'-(6-methylquinolinyl)]pyridine (0.018 g, 0.05 mmol), and ferrous chloride tetrahydrate (0.02 g, 0.05 mmol) along with five drops of 1 *M* hydrochloric acid were dissolved in ethanol (10 ml). The mixture was heated in a Teflon-lined, stainless-steel Parr bomb at 363 K for 120 h. The bomb was cooled at 5 K per hour. Black crystals were isolated.

### Refinement

Carbon-bound H-atoms were placed in calculated positions (C—H 0.95–0.98 Å) and were included in the refinement in the riding model approximation, with *U*(H) set to 1.2–1.5*U*(C).

One of the chlorine atoms is disordered over two positions in a 0.938 (11):0.062 (11) ratio. The Fe–Cl pair of distances were restrained to within 0.01 Å of each other; the anisotropic temperature factors of the minor component were restrained to be nearly isotropic.

### Crystal data

**Table:**

[FeCl_2_(C_25_H_19_N_3_)]	*Z* = 2
*M**_r_* = 488.18	*F*(000) = 500
Triclinic, *P*1	*D*_x_ = 1.552 Mg m^−^^3^
Hall symbol: -P 1	Mo *K*α radiation, λ = 0.71073 Å
*a* = 9.6228 (7) Å	Cell parameters from 4559 reflections
*b* = 10.2558 (8) Å	θ = 2.6–28.2°
*c* = 10.7324 (8) Å	µ = 1.00 mm^−^^1^
α = 94.352 (1)°	*T* = 100 K
β = 95.481 (1)°	Block, black
γ = 96.121 (1)°	0.30 × 0.10 × 0.05 mm
*V* = 1044.35 (14) Å^3^	

### Data collection

**Table:**

Bruker SMART APEX diffractometer	4757 independent reflections
Radiation source: fine-focus sealed tube	3954 reflections with *I* > 2σ(*I*)
graphite	*R*_int_ = 0.024
ω scans	θ_max_ = 27.5°, θ_min_ = 2.1°
Absorption correction: multi-scan (*SADABS*; Sheldrick, 1996)	*h* = −12→12
*T*_min_ = 0.754, *T*_max_ = 0.952	*k* = −13→13
9910 measured reflections	*l* = −13→13

### Refinement

**Table:**

Refinement on *F*^2^	Primary atom site location: structure-invariant direct methods
Least-squares matrix: full	Secondary atom site location: difference Fourier map
*R*[*F*^2^ > 2σ(*F*^2^)] = 0.035	Hydrogen site location: inferred from neighbouring sites
*wR*(*F*^2^) = 0.108	H-atom parameters constrained
*S* = 1.05	*w* = 1/[σ^2^(*F*_o_^2^) + (0.0511*P*)^2^ + 1.0201*P*] where *P* = (*F*_o_^2^ + 2*F*_c_^2^)/3
4757 reflections	(Δ/σ)_max_ = 0.001
292 parameters	Δρ_max_ = 0.54 e Å^−^^3^
7 restraints	Δρ_min_ = −0.44 e Å^−^^3^

### Fractional atomic coordinates and isotropic or equivalent isotropic displacement parameters (Å^2^)

**Table:**

	*x*	*y*	*z*	*U*_iso_*/*U*_eq_	Occ. (<1)
Fe1	0.34748 (3)	0.81412 (3)	0.22283 (3)	0.01918 (11)	
Cl1	0.16748 (18)	0.73795 (9)	0.34366 (18)	0.0225 (4)	0.938 (11)
Cl1'	0.211 (5)	0.730 (2)	0.377 (3)	0.047 (6)	0.062 (11)
Cl2	0.27672 (8)	0.85227 (6)	0.02182 (6)	0.03212 (17)	
N1	0.4298 (2)	0.61956 (18)	0.19090 (18)	0.0185 (4)	
N2	0.5602 (2)	0.84553 (19)	0.29589 (18)	0.0188 (4)	
N3	0.3828 (2)	1.02324 (19)	0.31020 (18)	0.0185 (4)	
C1	0.3563 (2)	0.5061 (2)	0.1319 (2)	0.0197 (5)	
C2	0.2114 (3)	0.5039 (2)	0.0943 (2)	0.0236 (5)	
H2	0.1656	0.5803	0.1097	0.028*	
C3	0.1373 (3)	0.3912 (2)	0.0355 (2)	0.0233 (5)	
H3	0.0398	0.3910	0.0107	0.028*	
C4	0.2005 (3)	0.2747 (2)	0.0101 (2)	0.0214 (5)	
C5	0.3407 (3)	0.2756 (2)	0.0458 (2)	0.0218 (5)	
H5	0.3846	0.1984	0.0289	0.026*	
C6	0.4219 (3)	0.3901 (2)	0.1079 (2)	0.0201 (5)	
C7	0.1113 (3)	0.1550 (2)	−0.0542 (2)	0.0268 (5)	
H7A	0.1709	0.0855	−0.0718	0.040*	
H7B	0.0637	0.1775	−0.1333	0.040*	
H7C	0.0411	0.1239	0.0004	0.040*	
C8	0.5659 (3)	0.3955 (2)	0.1470 (2)	0.0251 (5)	
H8	0.6136	0.3202	0.1327	0.030*	
C9	0.6372 (3)	0.5093 (3)	0.2057 (2)	0.0255 (5)	
H9	0.7346	0.5133	0.2328	0.031*	
C10	0.5660 (2)	0.6202 (2)	0.2256 (2)	0.0187 (5)	
C11	0.6408 (2)	0.7465 (2)	0.2875 (2)	0.0183 (5)	
C12	0.7816 (2)	0.7639 (2)	0.3330 (2)	0.0209 (5)	
H12	0.8377	0.6935	0.3254	0.025*	
C13	0.8396 (3)	0.8867 (2)	0.3902 (2)	0.0228 (5)	
H13	0.9361	0.9009	0.4218	0.027*	
C14	0.7556 (3)	0.9881 (2)	0.4005 (2)	0.0224 (5)	
H14	0.7932	1.0722	0.4401	0.027*	
C15	0.6156 (2)	0.9643 (2)	0.3520 (2)	0.0197 (5)	
C16	0.5151 (3)	1.0641 (2)	0.3564 (2)	0.0203 (5)	
C17	0.5577 (3)	1.1949 (3)	0.4088 (3)	0.0285 (6)	
H17	0.6527	1.2214	0.4412	0.034*	
C18	0.4599 (3)	1.2824 (3)	0.4121 (2)	0.0281 (6)	
H18	0.4876	1.3704	0.4466	0.034*	
C19	0.3201 (3)	1.2440 (2)	0.3654 (2)	0.0211 (5)	
C20	0.2134 (3)	1.3296 (2)	0.3667 (2)	0.0238 (5)	
H20	0.2370	1.4186	0.3999	0.029*	
C21	0.0778 (3)	1.2866 (2)	0.3213 (2)	0.0259 (5)	
C22	0.0444 (3)	1.1538 (2)	0.2698 (2)	0.0249 (5)	
H22	−0.0495	1.1237	0.2364	0.030*	
C23	0.1446 (3)	1.0680 (2)	0.2674 (2)	0.0223 (5)	
H23	0.1192	0.9793	0.2339	0.027*	
C24	0.2846 (3)	1.1113 (2)	0.3143 (2)	0.0199 (5)	
C25	−0.0365 (3)	1.3765 (3)	0.3212 (3)	0.0342 (6)	
H25A	0.0059	1.4683	0.3288	0.051*	
H25B	−0.0911	1.3611	0.3923	0.051*	
H25C	−0.0985	1.3583	0.2425	0.051*	

### Atomic displacement parameters (Å^2^)

**Table:**

	*U*^11^	*U*^22^	*U*^33^	*U*^12^	*U*^13^	*U*^23^
Fe1	0.01616 (18)	0.01714 (17)	0.02359 (19)	0.00279 (12)	0.00055 (13)	−0.00168 (13)
Cl1	0.0196 (5)	0.0208 (4)	0.0263 (6)	−0.0002 (3)	0.0040 (4)	−0.0017 (3)
Cl1'	0.046 (10)	0.052 (8)	0.042 (9)	−0.001 (7)	0.013 (8)	−0.005 (6)
Cl2	0.0433 (4)	0.0305 (3)	0.0245 (3)	0.0176 (3)	0.0004 (3)	−0.0001 (2)
N1	0.0183 (10)	0.0174 (9)	0.0195 (10)	0.0013 (7)	0.0009 (8)	0.0012 (7)
N2	0.0170 (9)	0.0187 (9)	0.0206 (10)	0.0006 (7)	0.0048 (8)	0.0005 (7)
N3	0.0173 (10)	0.0197 (9)	0.0182 (9)	0.0014 (8)	0.0023 (7)	0.0001 (7)
C1	0.0218 (12)	0.0175 (10)	0.0200 (11)	0.0019 (9)	0.0032 (9)	0.0014 (9)
C2	0.0221 (12)	0.0200 (11)	0.0279 (13)	0.0032 (9)	0.0000 (10)	−0.0021 (9)
C3	0.0191 (12)	0.0218 (11)	0.0279 (13)	0.0002 (9)	0.0005 (10)	0.0012 (10)
C4	0.0274 (13)	0.0191 (11)	0.0171 (11)	0.0009 (9)	0.0009 (9)	0.0014 (9)
C5	0.0275 (13)	0.0181 (11)	0.0203 (12)	0.0049 (9)	0.0032 (10)	0.0005 (9)
C6	0.0242 (12)	0.0185 (11)	0.0187 (11)	0.0037 (9)	0.0049 (9)	0.0040 (9)
C7	0.0339 (14)	0.0191 (11)	0.0255 (13)	−0.0015 (10)	0.0006 (11)	−0.0012 (9)
C8	0.0268 (13)	0.0236 (12)	0.0263 (13)	0.0073 (10)	0.0046 (10)	0.0019 (10)
C9	0.0214 (12)	0.0284 (13)	0.0276 (13)	0.0068 (10)	0.0025 (10)	0.0022 (10)
C10	0.0202 (11)	0.0186 (11)	0.0178 (11)	0.0028 (9)	0.0035 (9)	0.0032 (9)
C11	0.0186 (11)	0.0206 (11)	0.0154 (11)	0.0007 (9)	0.0036 (9)	0.0006 (9)
C12	0.0182 (11)	0.0263 (12)	0.0183 (11)	0.0036 (9)	0.0014 (9)	0.0016 (9)
C13	0.0177 (11)	0.0302 (12)	0.0202 (12)	0.0010 (10)	0.0029 (9)	0.0022 (10)
C14	0.0222 (12)	0.0228 (11)	0.0208 (12)	−0.0021 (9)	0.0030 (9)	−0.0016 (9)
C15	0.0210 (12)	0.0206 (11)	0.0181 (11)	0.0030 (9)	0.0046 (9)	0.0006 (9)
C16	0.0229 (12)	0.0201 (11)	0.0189 (11)	0.0026 (9)	0.0065 (9)	0.0023 (9)
C17	0.0276 (13)	0.0292 (13)	0.0274 (13)	0.0009 (11)	0.0000 (11)	0.0007 (10)
C18	0.0343 (15)	0.0222 (12)	0.0273 (13)	−0.0010 (11)	0.0056 (11)	0.0012 (10)
C19	0.0256 (12)	0.0192 (11)	0.0187 (11)	0.0009 (9)	0.0054 (9)	0.0012 (9)
C20	0.0309 (13)	0.0181 (11)	0.0232 (12)	0.0039 (10)	0.0076 (10)	0.0003 (9)
C21	0.0285 (13)	0.0242 (12)	0.0281 (13)	0.0085 (10)	0.0100 (10)	0.0063 (10)
C22	0.0227 (12)	0.0248 (12)	0.0285 (13)	0.0036 (10)	0.0066 (10)	0.0056 (10)
C23	0.0241 (12)	0.0192 (11)	0.0242 (12)	0.0029 (9)	0.0053 (10)	0.0010 (9)
C24	0.0230 (12)	0.0187 (11)	0.0196 (11)	0.0039 (9)	0.0071 (9)	0.0028 (9)
C25	0.0296 (14)	0.0257 (13)	0.0502 (18)	0.0101 (11)	0.0092 (13)	0.0059 (12)

### Geometric parameters (Å, °)

**Table:**

Fe1—N1	2.2386 (19)	C9—H9	0.9500
Fe1—N2	2.103 (2)	C10—C11	1.488 (3)
Fe1—N3	2.2523 (19)	C11—C12	1.384 (3)
Fe1—Cl1	2.3636 (8)	C12—C13	1.393 (3)
Fe1—Cl1'	2.369 (9)	C12—H12	0.9500
Fe1—Cl2	2.2748 (7)	C13—C14	1.388 (3)
N1—C10	1.328 (3)	C13—H13	0.9500
N1—C1	1.372 (3)	C14—C15	1.386 (3)
N2—C11	1.345 (3)	C14—H14	0.9500
N2—C15	1.349 (3)	C15—C16	1.483 (3)
N3—C16	1.333 (3)	C16—C17	1.416 (3)
N3—C24	1.376 (3)	C17—C18	1.369 (4)
C1—C2	1.411 (3)	C17—H17	0.9500
C1—C6	1.423 (3)	C18—C19	1.394 (4)
C2—C3	1.368 (3)	C18—H18	0.9500
C2—H2	0.9500	C19—C24	1.422 (3)
C3—C4	1.418 (3)	C19—C20	1.420 (3)
C3—H3	0.9500	C20—C21	1.363 (4)
C4—C5	1.365 (3)	C20—H20	0.9500
C4—C7	1.501 (3)	C21—C22	1.422 (4)
C5—C6	1.421 (3)	C21—C25	1.508 (3)
C5—H5	0.9500	C22—C23	1.373 (3)
C6—C8	1.403 (3)	C22—H22	0.9500
C7—H7A	0.9800	C23—C24	1.406 (3)
C7—H7B	0.9800	C23—H23	0.9500
C7—H7C	0.9800	C25—H25A	0.9800
C8—C9	1.365 (4)	C25—H25B	0.9800
C8—H8	0.9500	C25—H25C	0.9800
C9—C10	1.402 (3)		
			
N2—Fe1—N1	74.57 (7)	C10—C9—H9	120.2
N2—Fe1—N3	74.29 (7)	N1—C10—C9	122.6 (2)
N1—Fe1—N3	148.58 (7)	N1—C10—C11	116.18 (19)
N2—Fe1—Cl2	121.77 (6)	C9—C10—C11	121.2 (2)
N1—Fe1—Cl2	100.57 (5)	N2—C11—C12	121.4 (2)
N3—Fe1—Cl2	99.20 (5)	N2—C11—C10	114.5 (2)
N2—Fe1—Cl1	122.35 (8)	C12—C11—C10	124.1 (2)
N1—Fe1—Cl1	95.84 (6)	C11—C12—C13	118.8 (2)
N3—Fe1—Cl1	97.19 (6)	C11—C12—H12	120.6
Cl2—Fe1—Cl1	115.88 (6)	C13—C12—H12	120.6
N2—Fe1—Cl1'	109.4 (15)	C14—C13—C12	119.6 (2)
N1—Fe1—Cl1'	90.5 (9)	C14—C13—H13	120.2
N3—Fe1—Cl1'	95.9 (5)	C12—C13—H13	120.2
Cl2—Fe1—Cl1'	128.8 (15)	C15—C14—C13	118.6 (2)
Cl1—Fe1—Cl1'	13.1 (14)	C15—C14—H14	120.7
C10—N1—C1	118.53 (19)	C13—C14—H14	120.7
C10—N1—Fe1	114.81 (15)	N2—C15—C14	121.5 (2)
C1—N1—Fe1	126.46 (15)	N2—C15—C16	114.5 (2)
C11—N2—C15	120.0 (2)	C14—C15—C16	124.0 (2)
C11—N2—Fe1	119.82 (16)	N3—C16—C17	122.2 (2)
C15—N2—Fe1	120.19 (15)	N3—C16—C15	116.2 (2)
C16—N3—C24	118.6 (2)	C17—C16—C15	121.5 (2)
C16—N3—Fe1	114.61 (15)	C18—C17—C16	119.0 (2)
C24—N3—Fe1	126.69 (15)	C18—C17—H17	120.5
N1—C1—C2	119.2 (2)	C16—C17—H17	120.5
N1—C1—C6	121.8 (2)	C17—C18—C19	120.9 (2)
C2—C1—C6	119.0 (2)	C17—C18—H18	119.6
C3—C2—C1	119.6 (2)	C19—C18—H18	119.6
C3—C2—H2	120.2	C18—C19—C24	117.1 (2)
C1—C2—H2	120.2	C18—C19—C20	123.7 (2)
C2—C3—C4	122.5 (2)	C24—C19—C20	119.2 (2)
C2—C3—H3	118.8	C21—C20—C19	121.3 (2)
C4—C3—H3	118.8	C21—C20—H20	119.3
C5—C4—C3	118.5 (2)	C19—C20—H20	119.3
C5—C4—C7	122.5 (2)	C20—C21—C22	118.7 (2)
C3—C4—C7	119.0 (2)	C20—C21—C25	122.0 (2)
C4—C5—C6	121.1 (2)	C22—C21—C25	119.3 (2)
C4—C5—H5	119.4	C23—C22—C21	121.6 (2)
C6—C5—H5	119.4	C23—C22—H22	119.2
C8—C6—C5	123.2 (2)	C21—C22—H22	119.2
C8—C6—C1	117.5 (2)	C22—C23—C24	120.1 (2)
C5—C6—C1	119.4 (2)	C22—C23—H23	120.0
C4—C7—H7A	109.5	C24—C23—H23	120.0
C4—C7—H7B	109.5	N3—C24—C23	118.8 (2)
H7A—C7—H7B	109.5	N3—C24—C19	122.2 (2)
C4—C7—H7C	109.5	C23—C24—C19	119.0 (2)
H7A—C7—H7C	109.5	C21—C25—H25A	109.5
H7B—C7—H7C	109.5	C21—C25—H25B	109.5
C9—C8—C6	119.8 (2)	H25A—C25—H25B	109.5
C9—C8—H8	120.1	C21—C25—H25C	109.5
C6—C8—H8	120.1	H25A—C25—H25C	109.5
C8—C9—C10	119.7 (2)	H25B—C25—H25C	109.5
C8—C9—H9	120.2		
			
N2—Fe1—N1—C10	−3.07 (16)	C1—N1—C10—C11	179.1 (2)
N3—Fe1—N1—C10	−10.9 (2)	Fe1—N1—C10—C11	3.8 (3)
Cl2—Fe1—N1—C10	117.20 (16)	C8—C9—C10—N1	0.8 (4)
Cl1—Fe1—N1—C10	−125.06 (17)	C8—C9—C10—C11	−179.1 (2)
Cl1'—Fe1—N1—C10	−113.1 (13)	C15—N2—C11—C12	−1.4 (3)
N2—Fe1—N1—C1	−177.9 (2)	Fe1—N2—C11—C12	179.14 (17)
N3—Fe1—N1—C1	174.20 (17)	C15—N2—C11—C10	178.9 (2)
Cl2—Fe1—N1—C1	−57.66 (19)	Fe1—N2—C11—C10	−0.5 (3)
Cl1—Fe1—N1—C1	60.08 (19)	N1—C10—C11—N2	−2.3 (3)
Cl1'—Fe1—N1—C1	72.0 (13)	C9—C10—C11—N2	177.5 (2)
N1—Fe1—N2—C11	1.86 (17)	N1—C10—C11—C12	178.0 (2)
N3—Fe1—N2—C11	177.61 (19)	C9—C10—C11—C12	−2.1 (4)
Cl2—Fe1—N2—C11	−91.17 (17)	N2—C11—C12—C13	0.9 (3)
Cl1—Fe1—N2—C11	89.00 (17)	C10—C11—C12—C13	−179.5 (2)
Cl1'—Fe1—N2—C11	86.8 (7)	C11—C12—C13—C14	0.2 (4)
N1—Fe1—N2—C15	−177.57 (19)	C12—C13—C14—C15	−0.7 (4)
N3—Fe1—N2—C15	−1.81 (17)	C11—N2—C15—C14	0.9 (3)
Cl2—Fe1—N2—C15	89.40 (18)	Fe1—N2—C15—C14	−179.70 (18)
Cl1—Fe1—N2—C15	−90.42 (18)	C11—N2—C15—C16	−179.2 (2)
Cl1'—Fe1—N2—C15	−92.6 (7)	Fe1—N2—C15—C16	0.2 (3)
N2—Fe1—N3—C16	3.33 (16)	C13—C14—C15—N2	0.2 (4)
N1—Fe1—N3—C16	11.2 (2)	C13—C14—C15—C16	−179.7 (2)
Cl2—Fe1—N3—C16	−117.23 (16)	C24—N3—C16—C17	−0.3 (3)
Cl1—Fe1—N3—C16	124.98 (17)	Fe1—N3—C16—C17	176.64 (19)
Cl1'—Fe1—N3—C16	111.9 (15)	C24—N3—C16—C15	178.7 (2)
N2—Fe1—N3—C24	−180.0 (2)	Fe1—N3—C16—C15	−4.3 (3)
N1—Fe1—N3—C24	−172.12 (17)	N2—C15—C16—N3	2.9 (3)
Cl2—Fe1—N3—C24	59.44 (19)	C14—C15—C16—N3	−177.2 (2)
Cl1—Fe1—N3—C24	−58.34 (19)	N2—C15—C16—C17	−178.1 (2)
Cl1'—Fe1—N3—C24	−71.4 (15)	C14—C15—C16—C17	1.8 (4)
C10—N1—C1—C2	−180.0 (2)	N3—C16—C17—C18	−0.1 (4)
Fe1—N1—C1—C2	−5.3 (3)	C15—C16—C17—C18	−179.0 (2)
C10—N1—C1—C6	0.4 (3)	C16—C17—C18—C19	0.4 (4)
Fe1—N1—C1—C6	175.07 (17)	C17—C18—C19—C24	−0.3 (4)
N1—C1—C2—C3	−179.9 (2)	C17—C18—C19—C20	179.6 (2)
C6—C1—C2—C3	−0.3 (4)	C18—C19—C20—C21	−179.2 (2)
C1—C2—C3—C4	0.0 (4)	C24—C19—C20—C21	0.7 (4)
C2—C3—C4—C5	−0.1 (4)	C19—C20—C21—C22	−1.1 (4)
C2—C3—C4—C7	179.7 (2)	C19—C20—C21—C25	−179.6 (2)
C3—C4—C5—C6	0.5 (4)	C20—C21—C22—C23	1.3 (4)
C7—C4—C5—C6	−179.3 (2)	C25—C21—C22—C23	179.9 (2)
C4—C5—C6—C8	179.6 (2)	C21—C22—C23—C24	−1.2 (4)
C4—C5—C6—C1	−0.7 (4)	C16—N3—C24—C23	−179.1 (2)
N1—C1—C6—C8	0.0 (4)	Fe1—N3—C24—C23	4.4 (3)
C2—C1—C6—C8	−179.7 (2)	C16—N3—C24—C19	0.4 (3)
N1—C1—C6—C5	−179.7 (2)	Fe1—N3—C24—C19	−176.16 (17)
C2—C1—C6—C5	0.6 (3)	C22—C23—C24—N3	−179.8 (2)
C5—C6—C8—C9	179.7 (2)	C22—C23—C24—C19	0.8 (4)
C1—C6—C8—C9	0.1 (4)	C18—C19—C24—N3	−0.1 (4)
C6—C8—C9—C10	−0.4 (4)	C20—C19—C24—N3	−180.0 (2)
C1—N1—C10—C9	−0.7 (3)	C18—C19—C24—C23	179.4 (2)
Fe1—N1—C10—C9	−176.04 (19)	C20—C19—C24—C23	−0.5 (3)

### Hydrogen-bond geometry (Å, °)

**Table:**

*D*—H···*A*	*D*—H	H···*A*	*D*···*A*	*D*—H···*A*
C8—H8···Cl2^i^	0.95	2.70	3.561 (2)	151
C17—H17···Cl1^ii^	0.95	2.73	3.538 (4)	144

Symmetry codes: (i) −*x*+1, −*y*+1, −*z*; (ii) −*x*+1, −*y*+2, −*z*+1.
